# Root Causes of Poor Immunisation Data Quality and Proven Interventions: A Systematic Literature Review

**Published:** 2017-02-27

**Authors:** Olivia Wetherill, Chung-won Lee, Vance Dietz

**Affiliations:** 1 Monitoring & Evaluation, Policy and Performance, Gavi, The Vaccine Alliance, Switzerland; 2 Immunization Services Division, Centers for Disease Control and Prevention, USA

## Abstract

**Introduction:**

Effective allocation of resources and investments heavily rely on good quality data. As global investments in vaccines increases, particularly by organisations such as Gavi, The Vaccine Alliance, Switzerland, the demand for data which is accurate and representative is urgent. Understanding what causes poor immunisation data and how to address these problems are therefore key in maximizing investments, improving coverage and reducing risks of outbreaks.

**Objective:**

Identify the root causes of poor immunisation data quality and proven solutions for guiding future data quality interventions.

**Methods and Results:**

Qualitative systematic review of both scientific and grey literature using key words on immunisation and health information systems. Once screened, articles were classified either as identifying root causes of poor data quality or as an intervention to improve data quality. A total of 8,646 articles were initially identified which were screened and reduced to 26. Results were heterogeneous in methodology, settings and conclusions with a variation of outcomes. Key themes were underperformance in health facilities and limited Human Resource (HR) capacity at the peripheral level leading to data of poor quality. Repeated reference to a “culture” of poor data collection, reporting and use in low-income countries was found implying that it is the attitudes and subsequent behaviour of staff that prevents good quality data. Documented interventions mainly involved implementing Information Communication Technology (ICT) at the district level. However, without changes in HR capacity the skills and practices of staff remain a key impediment to reaching its full impact.

**Discussion:**

There was a clear incompatibility between identified root causes, mainly being behavioural and organizational factors, and interventions introducing predominantly technical factors. More emphasis should be placed on interventions that build on current practices and skills in a gradual process in order to be more readily adopted by health workers. Major gaps in the literature exist mainly in the lack of assessment at central and intermediate levels and association between inaccurate target setting from outdated census data and poor data quality as well as limited documentation of interventions that target behaviour change and policy change. This prevents the ability to make informed decisions on best methodology for improving data quality.

## Introduction

Data quality plays a fundamental role in the success or failure of an immunisation programme [1]. Poor quality immunisation data threatens to undermine national and international investments, prevents accurate monitoring of global immunization initiatives, and can increase the risk of vaccine preventable diseases (VPD) outbreaks by failing to identify areas or populations with low vaccination coverage [2]. The Global Vaccine Action Plan (GVAP) recognises poor data quality as a major obstacle in reaching the Decade of Vaccines mission as illustrated by its emphasis on strengthening immunisation systems [3]. Taking into consideration that 32 of 73 Gavi, The Vaccine Alliance, Switzerland, (Gavi) eligible countries had more than 10% difference between administrative and survey national DTP3 coverage estimates in the years 2011–2015 [4], Gavi, The Vaccine Alliance, Switzerland aims to provide support to countries on improving data availability, quality and use as part of their 2016–2020 strategy [5]. Additionally, all countries applying for support to Gavi, The Vaccine Alliance, Switzerland must prove data quality requirements have been met such as annual desk reviews, periodic in-depth data system assessments, regular surveys and a data improvement plan. Partnerships like the Health Data Collaborative highlight the global donor community’s enthusiasm to support health management information system (HMIS) strengthening through financial and technical advocacy [6]. It is therefore evident that more focus must be placed on assessing and implementing a strategic approach to improve immunisation data quality and mitigate the risks associated with poor data recording, reporting and use.

There are two common sources of national immunisation coverage data; national routine administrative data reporting system and household survey. These two sources of information regularly show discrepancies in immunisation coverage estimates, particularly in low-income settings [7]. For a typical health information system (HIS), the routine administrative information process involves data collection at the health facility level which is then transferred to district, regional and national offices sequentially for aggregation. Peripheral units in a low-income setting tend to employ a paper-based system with computerised systems and electronic databases at the more central levels. For data on vaccinations, national coverage data is submitted annually through the Joint Reporting Form (JRF) to World Health Organisation (WHO)/United Nations Children’s Emergency Fund (UNICEF) where analysis and discussions with corresponding countries on data quality follows [8]. The quality of immunisation data is affected by the strength of the routine health information system consisting of governance, workforce capacity and infrastructure and organisation. In addition to routine HIS, many countries conduct periodic household surveys, such as the Demographic and Health Surveys (DHS) and the Expanded Programme on Immunisations (EPI) coverage survey. Household surveys are usually viewed as more accurate than administrative data if a stringent and standardised methodology is employed. Despite this, however, there are instances of inconsistencies across household surveys of the same year [9]. While surveys can provide additional information that cannot be obtained by administrative data, such as linking coverage figures with demographic information to identify high risk groups, they are costly, resource intensive and time consuming. Additionally, there can be fundamental weaknesses in methodology such as dependence on mothers’ recall, selection bias and human errors in recording information [10,11].

Despite the global recognition for the need to improve and strengthen data quality, little is known on how to accomplish this goal. In addition to the limited literature on investigations to identify the root causes of poor data quality, there are also no standardised interventions that have proven to deliver sustainable and effective solutions. We therefore conducted a review of published and unpublished literature to compile the available evidence on the determinants of and effective interventions for poor data quality.

## Material and Methods

This review focused on the following key questions:

1. What are the root causes of poor immunisation data quality in Gavi, The Vaccine Alliance, Switzerland eligible countries?
2. What interventions can prevent these problems?

PubMed, Science Direct and Mendeley were used for searching the scientific literature. In addition Better Immunisation Data Intitiative, PATH, WHO, Gavi, The Vaccine Alliance, Switzerland, DHS Program, USAID, Unicef, Bill and Melinda Gates Foundation and MEASURE Evaluation websites were searched for grey literature. In order to capture a range of sources the following key words were used: “Immunis/zation” OR “Vaccination” OR “Coverage” OR “Gavi, The Vaccine Alliance, Switzerland” OR “Health management information system” OR “Health information system” AND “Data” OR “Survey” AND “Quality” OR “Accuracy” OR “Consistency” OR “Data use” OR “Availability”.

Sources were screened systematically first by title, abstract and finally full article using the following exclusion criteria: 1) published before 1986, 2) not written in English, 3) Focusing on reporting of Adverse Events Following Immunisation (AEFI), non-routine vaccines, or data quality of vaccine stocks/immunisation activities. Only articles that explicitly measured standard indicators for immunisation/health data quality or factors that influence data quality were included. Indicators that were considered to be reflective of data quality were validity, accuracy, relevance, completeness and timeliness.

### Classification and analysis

Following the screening and selection process, articles were then categorised according to either root causes of poor immunisation data or interventions that improved data quality. Within these two categories, classification of articles was based on previously researched conceptual frameworks. Lippeveld et al. [12] theorise that a health information system (HIS) is comprised of the information process and management structures [12]. Building on this framework, Aqil et al. [13] Performance of Routine Information System Management (PRISM) incorporates technical, organizational and behavioural factors as key determinants in the processes, outputs and outcomes of health information systems [13]. A combined approach was employed using these existing theories to then guide the classification of articles. For identifying root causes, the following groupings were used: “governance”, “programme management” and “HR capacity and practices”. Governance consists of both policies and the HIS structure, reflective of the PRISM’s organisational factor. Additionally, HR capacity and practices involve the attitudes and skills of staff similar to the PRISM’s behavioural factors. Finally, programme management is determined by a combination of behavioural and organisational factors since its performance is a function of HIS infrastructure and managers’ competencies. Interventions were split into two groupings: “Health Information Systems/Information Communication Technology (ICT) Interventions” and “Programme Management Interventions”. Interventions that modify HIS/ICT operations fell under all three of PRISM’s factors because as technical factors are introduced, governance and workers’ skills also must be altered. Articles were analysed and subsequently ranked according to the methodology, generalizability, representativeness and baseline review for interventions. Results show the strongest articles at the top (Tables 1 and 2).

## Results

**Figure 1:**
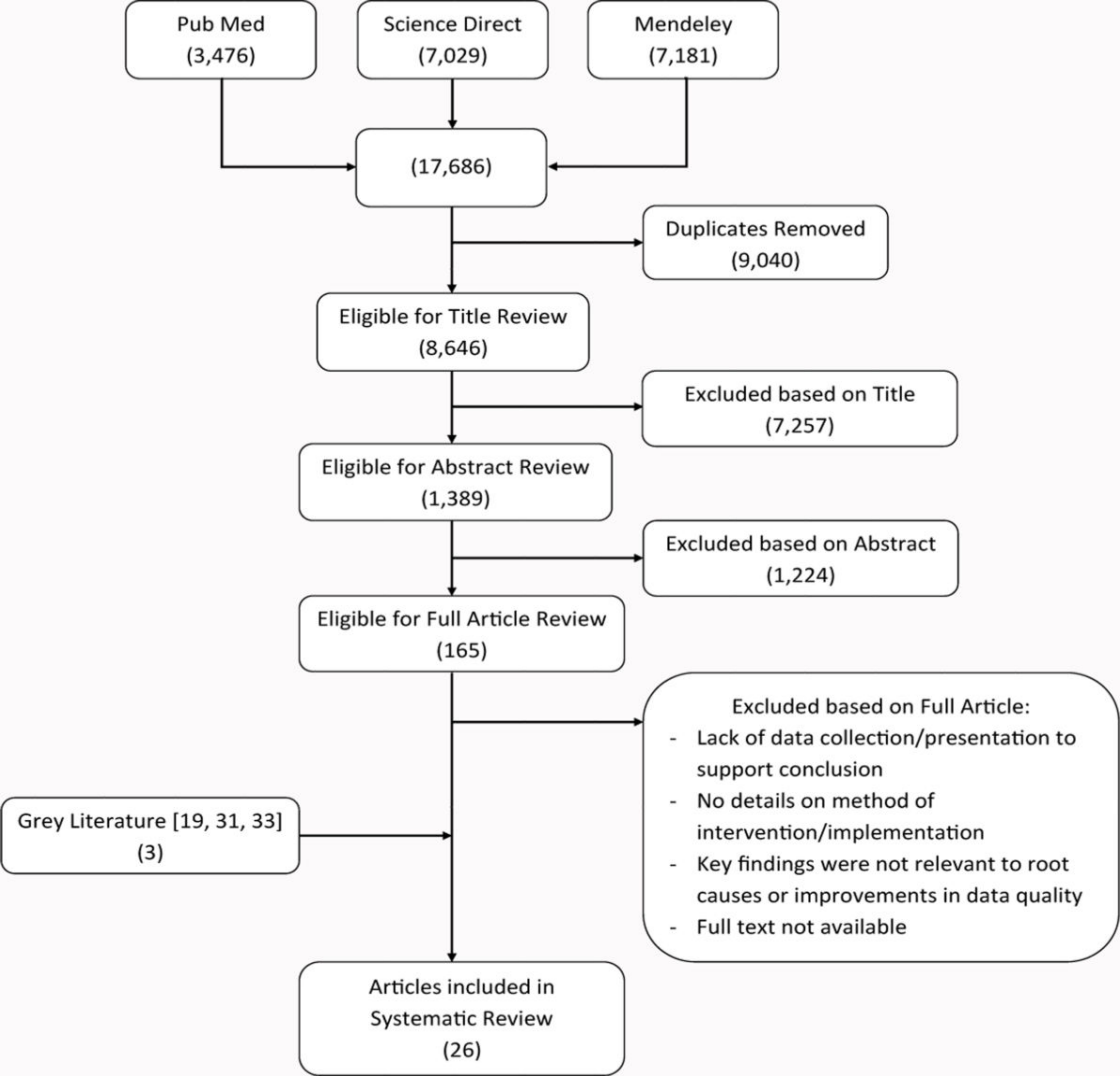
Flow chart of literature search and screening.

The search using PubMed, Science Direct and Mendeley identified a total of 8,646 (Figure 1). After screening using the inclusion/exclusion criteria and adding further relevant articles from references, a total of 26 articles were finalised for review. Due to heterogeneity in national reporting systems and for the purpose of creating uniformity in terminology, all facilities that immunised children in any setting are referred to as health facilities (HFs) and staff that immunise children and record immunization information at the health facility level are health workers (HWs).

Methodologies to identify and study root causes and to assess interventions were varied and utilized both quantitative and qualitative data collection. Consequently results were heterogeneous with conclusions that identified a variety of both root causes and varying outcomes of interventions. Out of the 16 articles that identified root causes, 11 directly measured data quality indicators [9,14–24] and 5 measured factors that influence data quality [25–28]. Of the 11 studies that directly assessed data quality, 6 compared figures between health facility records and district records [14,16,17,20,21,24], 1 compared home-based records to HF database records [23], 2 compared administrative estimates to survey estimates [9,15], 1 assessed data quality audits (DQAs) over time [18] and 1 analysed DHS survey data [19]. Of the five studies that did not measure data quality directly, three used key informant interviews [25–27] and 2 used formal surveys [19,28].

Although results were heterogeneous, key themes that emerged were the poor data handling practices and lack of HR capacity to record and collate data accurately at the peripheral levels where data collection originates. In addition, setting a target, either by incentivizing through performance based funding (PBF) or threatening to reduce salaries if targets are not met, was associated with over-reporting [15,17]. Studies also reported that unclear or culturally incompatible or insensitive policy-making and implementation is associated with staff disinterest resulting in inefficient data processing [25,26].

Among the studies assessing interventions, 4 conducted a baseline/control assessment of data quality to provide a comparison [29–31]. Of these studies 2 collected both quantitative and qualitative data [32,33] and 8 collected only quantitative data [18,29–31,34–36]. The majority of interventions focused on introducing technical approaches, using HIS and ICT, and were found to have mixed results [29–37]. Reasons for implementing these technical interventions were predominantly to improve and streamline data processes, for example by upgrading methods from a paper-based system, and to provide opportunity for data analysis at the local level. Areas where this category of interventions failed to reach their full potential mainly were attributed to human errors when using technology due to insufficient data analysis skills, and the lack of or poor supervision [29,34–36]. One articles focused on improving the management of data processing, both showed improvements in data quality as measured by the above mentioned indicators [38].

## Discussion

Inaccurate and unreliable data can significantly impair the ability to make evidence-based decision making [39]. Because the infrastructure, governance and practices of an HIS directly influence data quality, it is thought that improvements in data should be part of the effort to strengthen health systems as a whole with improved quality of data enhancing better decision-making. We aimed to identify root causes of poor data quality and interventions that can provide effective and sustainable solutions to these problems.

Establishing a causal link between a determinant and data quality is challenging particularly as there are many different components of data management within an HIS that can influence the final quality of immunization data. Due to this complexity, classification of some articles were not wholly representative of its reported root cause. For example, the instance where incomplete information in the healthcare database was due to the high workload of staff, this problem can be attributed to a variety of reasons [23]. It may be that financial strain on the health system results in reduced staff numbers and high workload, that the data processing by design causes too much workload or that programme managers have not effectively allocated responsibilities to allow for efficient data collection.

Comparing figures in different stages of data processing, e.g., between HF reports and district reports, sheds light on where discrepancies may arise. However this does not explain from where the root causes of these discrepancies arise. In addition, this method of comparison can be resource intensive and the majority of articles reviewed applied this methodology in only a small number of districts and health facilities bringing into question the generalizability of the results. Another methodology, employed by three articles [20,21,24], used an adaptation of the World Health Organisation Data Quality Self-assessment tool [40]. Whilst this method is useful for identifying areas for improvement, an inherent fault lies in the lack of data validity with survey information [41]. Articles that indirectly measured data quality through key informant interviews and formal surveys [19,25–28] were helpful for identifying the role of attitudes and skills of staff, however provided limited proof that these factors directly caused poor data quality.

The literature reviewed indicates that impediments to achieving a high standard of data quality is predominantly located at the peripheral levels due to HR capacity shortages and the skills of staff. This corresponds with other studies that have highlighted the weak skills and inadequate application of data utilisation for decision making at the local level [42,43]. There is however limited evidence to suggest whether these inadequate data handling practices also occur at the central and intermediate levels.

As well as problems with staff abilities, issues in the management structures of HIS can also influence data quality. For example lack of clear guidelines on data validation protocol will prevent effective checking of information [14]. Additionally, policies involving target setting increases chances of over-reporting [15]. For calculating targets at the local level, outdated census data is a major obstacle. We were unable to find any literature that explicitly measured the link between inaccurate population measures and subsequent target setting to result in poor data quality. More research and guidance is therefore needed to prioritise this issue.

Only 4 articles measured quantifiable changes in data quality post-intervention using a baseline or control group thus highlighting the shortage in evidence-based interventions [29–31,38]. Kiberu et al. [29], however, used baseline data from the newly implemented DHIS2, rather than raw pre-intervention data, bringing into question the methodology.

Whilst identified root causes mainly fell under the PRISM’s behavioural and organizational factors at the peripheral level, there is clear incompatibility with interventions since the majority focused on introducing technical factors such as ICT interventions at the district level. Although the main justification for these interventions was to provide technology that would upgrade and streamline data processes, the capacities and practices at the HF level were bypassed thereby preventing maximum use of technology and its beneficial impact. Even at the district level, simply introducing a technology with no appropriately trained staff does not ensure gains in data quality and can jeorpardise its sustainability [44]. Two interventions that clearly demonstrated measured improvements in data quality attributed their success to either the ongoing intervention activities and associated monitoring through DQAs [30] or the reallocation of aggregation tasks to community health workers after baseline assessment [38]. Both of these interventions had collected information on prior knowledge of data processes and built on this to introduce incremental changes that could be easily adopted by health workers. This method of developing step-by-step interventions based on already learnt skills, as opposed to a ‘design from nowhere’ approach, results in sustainable solutions and solid foundation from which to develop effective scaling up of information systems [45].

Despite the major gaps in the literature, this review has highlighted how data quality is influenced by a multitude of factors. A broad term that is commonly cited as a major impediment to effective HIS operation is a ‘culture’ of poor data practices and information use, implying the fundamental issues lie in staff attitude and subsequent behaviour. The origins of data culture is virtually impossible to understand but it can be assumed that the initial design of an HIS with no end-user consideration together with ineffective management and staff training instigates and prolongs a poor attitude to data amongst workers. The mindset that data is merely a means of passing on information rather than an end in itself is particularly discouraging as HISs become more decentralised [46]. Unfortunately, mechanisms to address this issue are poorly understood or if implemented, often do not succeed. For example, persuading data use through workshops in Zanzibar proved to be effective in facilitating discussion around HMIS developments however there was no evidence to validate whether this actually resulted in improved data quality [47].

Human behaviour change is one of the most challenging aspects of an intervention but one that can provide truly sustainable results. An approach that is flexible and integrated, not only intervening at the local level but also taking advantage of the impact of policy implementation, is recognised as the future of data quality interventions [48]. However, more research must be conducted that evaluates and documents how interventions targeting behavioural and organisational factors affects the quality of health information systems.

## Figures and Tables

**Table 1:** Root causes of poor health data quality reported through published and unpublished literature 1985–2015.

Category 1 Root Causes: Policy & Governance				
Objective & Setting	Methodology	Key Findings	Root Causes Interpreted	Reference
Evaluation of immunisation data quality using qualitative and quantitative methods in selected districts in Mozambique	Key Informant Interviews (KII), Observations (O), Desk Review (DR)	Data inconsistencies were found between tally sheets and HF reports as well as between HF reports and district reports, poor validation method by supervision and management feedback was based on criticism	Poor support system based on condemnation and lack of standards for data verification leads to inaccurate immunisation reporting	[14]
Measurement of the effect of Gavi, The Vaccine Alliance, Switzerland Immunisation Services Support (ISS) on immunisation data quality in Gavi-eligible countries	Analysis of admin coverage with survey coverage estimates	Presence of Gavi, The Vaccine Alliance, Switzerland ISS support is associated with wider disparities between admin and survey coverage estimates	Performance-based funding and political pressure encourages national over-reporting	[15]
Assessment of coverage estimates in Burkina Faso	Comparison of admin estimates with EPI and DHS survey estimates in 2003	Overestimation and underestimation occurs in rural districts and districts near urban areas respectively. National coverage estimates do not accurately reflect the heterogeneity of districts	Outdated and inconsistent denominator use reduces validity of admin data	[9]
Evaluation of HMIS data quality in Kinondoni district, Tanzania	Analysis of available HMIS reports from HF to district	There is a twofold variation for each step in data processing and significant incomplete reporting by the private health sector.	Ineffective recording system and validation together with poor governance of private sector impede accurate data reporting	[16]
Qualitative assessment of health managers’ perspective on HMIS use in Pakistan	KII	Although HMIS had proven sustainability and a good sense of ownership, managers remark on political interference, incomplete HMIS coverage and corruption within the system	Lack of transparency and unclear policy-making within HMIS prevents data skills development which in turn reduces the quality of data	[25]
Evaluation of District Health Information System (DHIS) data use for health programme management in Kenya	KII, Focus Group (FG), Formal Survey (FS), analysis of operational manuals	DHIS systems were fragmented with a lack of basic resources and poor data quality	Design of information system with no consideration of end users results in inefficient and unsustainable data processing	[26]
**Category 2 Root Causes: Programme Management**				
Assessment of data quality using “Data Entry via Phone Image Capture” (DEPIC) in Thailand	Comparison of home-based records database with HF electronic database	HF electronic information system was incomplete compared to DEPIC database with inconsistencies in vaccination dates	Poor data entry into electronic database is caused by delays due to excessive workload of staff on pre- scheduled immunisation days	[23]
Assessment of national immunisation data quality in Nepal	Comparison of immunisation register, HF and district reports	HF summary reports over-reported number of children immunised than those on the immunisation registers whilst district reports over and under-reported number immunised from HF reports	The pressure to reach immunisation targets from the central level outweighed the support for accurate and honest reporting	[17]
**Category 3 Root Causes: Hr Capacity & Practices**				
Assessment of immunisation data quality in 41 Gavi, The Vaccine Alliance, Switzerland eligible countries	Evaluation of Data Quality Audits between 2002–2005	Almost half countries ‘failed’ the DQA mostly due to poor feedback and inconsistent denominator calculations	Inadequate data practices at the peripheral level remain key determinants of poor data quality despite extensive guidelines and training	[18]
Assessment of DHS data quality and field related factors	Analysis of DHS data 2003–2006	Fieldwork conducted in rural areas and use of translator during interviews are highly associated with inaccurate data recording	Inadequate HR capacity can limit accuracy of survey data	[19]
Evaluation of immunisation data quality in 2 districts of Nigeria	Data Quality Self-assessment (DQS)	Data discrepancy between HF and district office was significant with hardly any monitoring or data use activities at the HF level	Lack of skills, feedback and knowledge at the HF level contributes and self-perpetuates a culture of poor data quality checking and use	[20]
Evaluation of immunsiation data quality in 8 sub-districts of Ghana	DQS, O	HF reports were consistently higher than HF tally sheets with no data quality improvement in time. Half of HFs also had incomplete tally books.	Inadequate data handling practices at facility level results in inconsistencies and over reporting	[21]
Evaluation of DHIS in rural South Africa	KII, DR of clinic data over 12 months	Despite accurate information collection, data quality and utilization remained poor with rare checking of data and no feedback from district to clinic supervisors	The lack of skills and understanding for data use at the facility level compromises the quality of data	[22]
Qualitative assessment of factors influencing HMIS in Tanzania	KII	Despite an overall positive attitude to HMIS, the majority of HWs had never been trained and understanding of HMIS was poor	Lack of understanding and skills amongst HWs leads to poor quality of data collection	[27]
Assessment of behavioural factors’ effect on quality of Routine Health Information System (RHIS) in South Africa	FS	Confidence was high but RHIS competence and data quality checking skills was low at all levels	Incompetent skills at HF and district level results in careless recording and reporting of health data	[28]
Evaluation of immunisation data quality in 2 districts of Tunisia	DQS	Data discrepancies were found across different data sources particularly at HF level. M&E and computerised recording of data were the poorest performing quality indicator at the HF and district office respectively.	Poor M&E practices at HF level together with complex recording protocol contribute to substandard recording and reporting	[24]

**Table 2:** Interventions to strengthen health data quality and relevant practices through published and unpublished literature 1985–2015.

Category 1 Interventions: Health Information System / Ict Interventions				
Study Objectives & Setting	Intervention	Assessment Methodology	Outcomes	Reference
Validation of Uruguayan National Immunisation Registry (NIR) for assessing immunisation coverage	National roll-out of NIR	Concordance analysis of immunisation numerator and denominator at HF, district and national levels across various sources	NIR improved the monitoring of coverage status/vaccination status however the data analysis and feedback is still weak.	[37]
Measurement of impact of DHIS2 on quality of coverage indicators in Uganda	National roll-out of DHIS2	Pre/post (1 year) comparison of timeliness and completeness of health service coverage indicators	Completeness and timeliness of reports improved however limited technical staff and high staff turnover created a deficiency in skilled personnel. Substandard recording at facility levels also persisted.	[29]
Assessment of HR capacity for the use of HIS in Tanzania	DHIS implementation and relevant trainings	KII, DR, O	Lack of culturally compatible training together with poor health manager participation are main factors for holding back data quality	[32]
Report on national roll-out of Kenyan DHIS2	Rapid national roll-out of DHIS2 through central server	KII, DR	Despite initial internet connectivity problems, DHIS2 increased report completeness and allowed more opportunity to analyse data.	[33]
Evaluation of the long-term impact of Population Health Implementation and Training (PHIT) Partnership in Mozambique	PHIT implementation and relevant trainings	Retrospective analysis of yearly DQAs over 3 years	Data concordance significantly increased and sustained at a relatively high level mainly due to ongoing intervention activities	[30]
Assessment of coverage and quality of ProMIS, a computerised RHIS in Mali	ProMIS implementation and relevant trainings	Data collected through mothers’ interviews, using LQAS methodology, were compared to data in ProMIS	Lack of staff and equipment, such as printers, together with inadequate technical assistance created backlog of data entry and loss of data use for decision-making	[34]
Evaluation of the use of handheld devices for the management of HMIS data in rural Tanzania	Distribution and training on handheld personal digital assistants (PDAs)	Comparison of data entries between PDA operator and data entry clerk	Even though the technology was durable, human errors made whilst recording outweighed the benefits of electronic data capture and quality did not improve	[35]
Report on implementation of a national electronic immunisation registry in Albania	Electronic immunization registry implementation and relevant trainings	Pre and post intervention comparison of coverage and cohort reports measuring completeness, accuracy, timeliness and relevance	Whilst data is more accurate with better internal consistency, implementing the system required regular feedback from users and building on already learnt skills	[31]
Report on national roll-out of revised HMIS in Kyrgyzstan	HMIS review and national roll-out	Assessment conducted using supervision checklists 5 months post national roll-out	Increase in accuracy and timeliness of reports and better sense of ownership over work however supervision still remained inadequate	[36]
**Category 2: Programme Management Interventions**				
Assessment of data quality after Health Information System (HIS) intervention	Reallocation of aggregation job and modification of reporting formats	LQAS methodology comparing reported and re-aggregated indicators	Data consistency between reported and re-aggregated numbers improved	[38]
